# Editorial: Atomic Clusters: Theory & Experiments

**DOI:** 10.3389/fchem.2021.795113

**Published:** 2021-11-01

**Authors:** Ambrish Kumar Srivastava, Iwona Anusiewicz, Suzana Velickovic, Wei-Ming Sun, Neeraj Misra

**Affiliations:** ^1^ Department of Physics, Deen Dayal Upadhyaya Gorakhpur University, Gorakhpur, India; ^2^ Department of Chemistry, University of Gdansk, Gdansk, Poland; ^3^ Department of Physical Chemistry, Vinĉa Institute of Nuclear Science, University of Belgrade, Belgrade, Serbia; ^4^ Department of Basic Chemistry, Fujian Medical University, Fuzhou, China; ^5^ Department of Physics, University of Lucknow, Lucknow, India

**Keywords:** atomic clusters, theory, superatom, fullerene, nanostructure

Atomic clusters are finite aggregates of atoms, varying in size from a few Angstrom to a few nanometers. The importance of atomic clusters lies in the fact that they possess very unique properties, which are sometimes, quite different than their bulk analogs. Due to advancements in theory and instrumentation along with the aid of powerful computers, the research and development in this field are greatly accelerated. The topic “*Atomic Clusters: Theory & Experiments*” provide a compilation of the recent progress made in this very exciting field of research. This topic consists of two review and seven research articles on various themes, which are outlined below.

Pal et al. reviewed the various aspects of atomic clusters. They discussed the structures of certain atomic clusters such as noble gas encapsulated B_40_ cage, small molecule encapsulated octa acid, (HF)_2_ confined fullerenes, etc. using different machine learning techniques. The bonding and reactivity of these clusters were discussed with the help of the quantum theory of atoms in molecule (QTAIM) and conceptual density functional theory (CDFT). Srivastava reviewed various optimization algorithms such as genetic algorithm (GA), basin-hopping (BH) method and its variants, heuristic algorithm combined with the surface and interior operators (HA-SIO), fast annealing evolutionary algorithm (FAEA), random tunneling algorithm (RTA), and dynamic lattice searching (DLS) to obtain the global minimum structures of the different type clusters such as pure metallic clusters, bimetallic clusters, trimetallic and tetrametallic clusters, fullerene-like clusters, and dipolar clusters.

Zhou et al. introduced a newly developed NKCS python code based on xTB local optimization and BH global search algorithm. They obtained global minimum structures of the cations of phosphorus clusters, P_2*n*+1_^+^ for *n* = 1–15 in which the pnicogen bonds play an important role in the stabilization of clusters and identified P_29_^+^ and P_31_^+^ as the most stable isomers. Their results showed that the NKCS program is effective and robust in searching global minimum structures for atomic clusters. Shi et al. searched the lowest-energy structures of hydrated calcium ion clusters Ca^2+^(H_2_O)_*n*_ (*n* = 10–18) in the whole potential energy surface by the comprehensive genetic algorithm (CGA) combined with DFT. Their theoretical results could provide useful guidance for analyzing the hydrated calcium ion clusters in experiments, and are of fundamental importance for an in-depth understanding of the microscopic interactions between Ca^2+^ and water molecules in aqueous environments.

Jiang et al. examined the stability of two transition metal boron clusters Sc_2_B_8_ and Y_2_B_8_ in the inverse sandwich configuration via first-principle calculations combined with a comprehensive genetic algorithm (CGA). It is confirmed that such novel structures are the lowest-energy isomers and can be extended to 1D nanowires (NWs). They revealed that both theoretically designed 1D-Sc_4_B_24_ and 1D-Y_2_B_12_ nanowires are nonmagnetic such that the former NW is a direct-band-gap semiconductor, whereas the latter one is a metal. Tiznado et al. investigated the stability of the isolated silicon-lithium nanowire (Li_6_Si_5_-NW) assembled from stacking the Li_6_Si_5_ units as well as its electronic properties by using DFT methods and Born-Oppenheimer *ab initio* molecular dynamic simulations. They studied the possibility of using carbon nanotubes (CNTs) as an alternative way to stabilize thus obtained 1D Li_6_Si_5_-NW by stacking Li_6_Si_5_ units one above another and confirmed its metallic character. They found that finite (Li_6_Si_5_)_4_ systems are stable inside both armchair and zigzag CNTs which supports the hypothesis of possible formation of Li_6_Si_5_-NW in CNTs.

Yu et al. reported an osmium-centered aromatic cluster of boron, OsB_9_^−^ using DFT and QTAIM approaches. They described the structure, energetics, electron delocalization as well as photoelectron spectrum. Their findings suggested that the dual *σ* + *π* aromaticity is a key factor to design highly stable borometallic molecular wheels. Meloni et al. studied Li_3_F_2_ superalkali encapsulated C_60_ fullerene by DFT and found that this endofullerene is stable. They noticed that the CO_2_ molecule can be activated by trapping within this endofullerene. During the activation, an F atom of Li_3_F_2_ is bonded to the CO_2_, unlike a simple electron transfer process. These findings suggested the activation of CO_2_ at the nanoscale. Qasemnazhand et al. investigated the structure of sila-fulleranes (Si_*n*_H_*n*_; *n* = 20–60) and the interaction of Si_20_H_20_ with glycoprotein. They compared the electronic absorption spectrum of pure Si_20_H_20_ with those interacting with glycoproteins through O- and N-links. They suggested that the optical response of sila-fullerane changes when it interacts with viral spikes and therefore, it acts as a sensor for monitoring the environment.

Thus, the topic covers the articles on a variety of themes such as non-metallic clusters, metallic clusters, nanowires, fullerenes, etc. and introduces the readers to the current status in this rapidly growing field of research. We, the editors, thank all the authors for contributing to this topic as well as reviewers for their voluntary support. We believe that the contents of the topic will benefit the scientific community at large.

